# Supplementary material for: Modeling glioblastoma heterogeneity as a dynamic network of cell states
Source: Mol Syst Biol. 2021 Sep 16;17(9):e10105. doi: 10.15252/msb.202010105 (PMC8444284; doi:10.15252/msb.202010105)
Supplement: Supplementary file 5 — Source Data for Figure 3 [file MSB-17-e10105-s001.zip › Figure3A_sourcedata/GSEA_3065/hallmarks_state6.GseaPreranked.1623416308702/HALLMARK_HEME_METABOLISM.html]

Details for gene set HALLMARK\_HEME\_METABOLISM[GSEA]

|  || Dataset | state6 |
| Phenotype | NoPhenotypeAvailable |
| Upregulated in class | na\_pos |
| GeneSet | HALLMARK\_HEME\_METABOLISM |
| Enrichment Score (ES) | 0.27028608 |
| Normalized Enrichment Score (NES) | 1.1715814 |
| Nominal p-value | 0.132626 |
| FDR q-value | 0.3439107 |
| FWER p-Value | 0.975 |
Table: GSEA Results Summary

  

Fig 1: Enrichment plot: HALLMARK\_HEME\_METABOLISM      
 Profile of the Running ES Score & Positions of GeneSet Members on the Rank Ordered List

  

| PROBE | GENE SYMBOL | GENE\_TITLE | RANK IN GENE LIST | RANK METRIC SCORE | RUNNING ES | CORE ENRICHMENT || 1 | BTG2 |  |  | 63 | 0.234 | 0.0240 | Yes |
| 2 | RBM38 |  |  | 178 | 0.183 | 0.0353 | Yes |
| 3 | BNIP3L |  |  | 240 | 0.164 | 0.0501 | Yes |
| 4 | HDGF |  |  | 247 | 0.162 | 0.0711 | Yes |
| 5 | GLRX5 |  |  | 368 | 0.141 | 0.0760 | Yes |
| 6 | NR3C1 |  |  | 377 | 0.139 | 0.0937 | Yes |
| 7 | TFDP2 |  |  | 507 | 0.122 | 0.0952 | Yes |
| 8 | MFHAS1 |  |  | 571 | 0.116 | 0.1035 | Yes |
| 9 | TCEA1 |  |  | 576 | 0.116 | 0.1185 | Yes |
| 10 | RAD23A |  |  | 622 | 0.112 | 0.1283 | Yes |
| 11 | MOSPD1 |  |  | 633 | 0.111 | 0.1420 | Yes |
| 12 | RIOK3 |  |  | 687 | 0.107 | 0.1501 | Yes |
| 13 | SMOX |  |  | 689 | 0.107 | 0.1643 | Yes |
| 14 | HEBP1 |  |  | 702 | 0.105 | 0.1770 | Yes |
| 15 | ADIPOR1 |  |  | 779 | 0.100 | 0.1815 | Yes |
| 16 | MGST3 |  |  | 824 | 0.097 | 0.1894 | Yes |
| 17 | ASNS |  |  | 850 | 0.094 | 0.1991 | Yes |
| 18 | HTRA2 |  |  | 907 | 0.091 | 0.2047 | Yes |
| 19 | YPEL5 |  |  | 925 | 0.090 | 0.2147 | Yes |
| 20 | BLVRB |  |  | 928 | 0.090 | 0.2265 | Yes |
| 21 | KAT2B |  |  | 941 | 0.089 | 0.2370 | Yes |
| 22 | TOP1 |  |  | 1024 | 0.084 | 0.2388 | Yes |
| 23 | PRDX2 |  |  | 1061 | 0.082 | 0.2456 | Yes |
| 24 | ARL2BP |  |  | 1096 | 0.081 | 0.2525 | Yes |
| 25 | GYPC |  |  | 1191 | 0.076 | 0.2518 | Yes |
| 26 | MXI1 |  |  | 1289 | 0.072 | 0.2502 | Yes |
| 27 | UROD |  |  | 1324 | 0.070 | 0.2556 | Yes |
| 28 | TRAK2 |  |  | 1335 | 0.070 | 0.2638 | Yes |
| 29 | SLC6A8 |  |  | 1391 | 0.068 | 0.2665 | Yes |
| 30 | ALDH6A1 |  |  | 1436 | 0.066 | 0.2703 | Yes |
| 31 | MKRN1 |  |  | 1685 | 0.057 | 0.2494 | No |
| 32 | FBXO7 |  |  | 1791 | 0.054 | 0.2444 | No |
| 33 | UBAC1 |  |  | 1797 | 0.054 | 0.2510 | No |
| 34 | BLVRA |  |  | 1813 | 0.053 | 0.2564 | No |
| 35 | CIR1 |  |  | 1826 | 0.053 | 0.2620 | No |
| 36 | EIF2AK1 |  |  | 1907 | 0.050 | 0.2596 | No |
| 37 | ISCA1 |  |  | 2007 | 0.048 | 0.2545 | No |
| 38 | UCP2 |  |  | 2041 | 0.047 | 0.2569 | No |
| 39 | FOXO3 |  |  | 2178 | 0.042 | 0.2469 | No |
| 40 | LPIN2 |  |  | 2188 | 0.042 | 0.2515 | No |
| 41 | NCOA4 |  |  | 2207 | 0.041 | 0.2549 | No |
| 42 | ADD2 |  |  | 2213 | 0.041 | 0.2598 | No |
| 43 | BTRC |  |  | 2282 | 0.039 | 0.2572 | No |
| 44 | DAAM1 |  |  | 2604 | 0.031 | 0.2243 | No |
| 45 | BACH1 |  |  | 2686 | 0.029 | 0.2188 | No |
| 46 | DCUN1D1 |  |  | 2882 | 0.024 | 0.1996 | No |
| 47 | SDCBP |  |  | 2964 | 0.023 | 0.1933 | No |
| 48 | VEZF1 |  |  | 3022 | 0.022 | 0.1896 | No |
| 49 | HMBS |  |  | 3229 | 0.017 | 0.1682 | No |
| 50 | FBXO34 |  |  | 3282 | 0.016 | 0.1643 | No |
| 51 | SLC10A3 |  |  | 3296 | 0.016 | 0.1649 | No |
| 52 | HAGH |  |  | 3373 | 0.014 | 0.1579 | No |
| 53 | UROS |  |  | 3426 | 0.012 | 0.1536 | No |
| 54 | TNRC6B |  |  | 3434 | 0.012 | 0.1544 | No |
| 55 | TMEM9B |  |  | 3494 | 0.011 | 0.1491 | No |
| 56 | NARF |  |  | 3540 | 0.011 | 0.1454 | No |
| 57 | NUDT4 |  |  | 3591 | 0.009 | 0.1409 | No |
| 58 | OPTN |  |  | 3659 | 0.008 | 0.1342 | No |
| 59 | PPOX |  |  | 3685 | 0.007 | 0.1322 | No |
| 60 | CAST |  |  | 3718 | 0.006 | 0.1294 | No |
| 61 | FBXO9 |  |  | 3826 | 0.004 | 0.1176 | No |
| 62 | GDE1 |  |  | 3856 | 0.004 | 0.1148 | No |
| 63 | BPGM |  |  | 3906 | 0.002 | 0.1094 | No |
| 64 | CAT |  |  | 3983 | 0.001 | 0.1008 | No |
| 65 | CCND3 |  |  | 4016 | 0.000 | 0.0972 | No |
| 66 | PSMD9 |  |  | 4231 | -0.004 | 0.0730 | No |
| 67 | OSBP2 |  |  | 4271 | -0.004 | 0.0691 | No |
| 68 | CPOX |  |  | 4647 | -0.012 | 0.0275 | No |
| 69 | MARK3 |  |  | 4659 | -0.012 | 0.0278 | No |
| 70 | SEC14L1 |  |  | 4793 | -0.014 | 0.0143 | No |
| 71 | CCDC28A |  |  | 4806 | -0.014 | 0.0149 | No |
| 72 | ALAD |  |  | 4826 | -0.015 | 0.0147 | No |
| 73 | P4HA2 |  |  | 4919 | -0.017 | 0.0063 | No |
| 74 | LRP10 |  |  | 5011 | -0.018 | -0.0017 | No |
| 75 | EPB41 |  |  | 5038 | -0.019 | -0.0021 | No |
| 76 | FECH |  |  | 5072 | -0.020 | -0.0033 | No |
| 77 | AGPAT4 |  |  | 5150 | -0.021 | -0.0093 | No |
| 78 | SLC25A38 |  |  | 5287 | -0.024 | -0.0218 | No |
| 79 | SLC7A11 |  |  | 5291 | -0.024 | -0.0189 | No |
| 80 | NFE2L1 |  |  | 5304 | -0.024 | -0.0171 | No |
| 81 | ARHGEF12 |  |  | 5476 | -0.028 | -0.0330 | No |
| 82 | PICALM |  |  | 5655 | -0.032 | -0.0493 | No |
| 83 | GCLM |  |  | 5804 | -0.034 | -0.0618 | No |
| 84 | USP15 |  |  | 5809 | -0.035 | -0.0576 | No |
| 85 | XPO7 |  |  | 5818 | -0.035 | -0.0539 | No |
| 86 | CA2 |  |  | 5950 | -0.037 | -0.0641 | No |
| 87 | GMPS |  |  | 6067 | -0.040 | -0.0722 | No |
| 88 | TFRC |  |  | 6511 | -0.050 | -0.1165 | No |
| 89 | CLCN3 |  |  | 6516 | -0.050 | -0.1103 | No |
| 90 | BCAM |  |  | 6610 | -0.053 | -0.1139 | No |
| 91 | PGLS |  |  | 6639 | -0.054 | -0.1100 | No |
| 92 | KLF3 |  |  | 6686 | -0.055 | -0.1079 | No |
| 93 | BMP2K |  |  | 6703 | -0.056 | -0.1023 | No |
| 94 | CDC27 |  |  | 6787 | -0.058 | -0.1040 | No |
| 95 | GAPVD1 |  |  | 6822 | -0.059 | -0.1000 | No |
| 96 | IGSF3 |  |  | 6865 | -0.061 | -0.0967 | No |
| 97 | RNF19A |  |  | 6915 | -0.062 | -0.0941 | No |
| 98 | ADD1 |  |  | 6959 | -0.063 | -0.0906 | No |
| 99 | MAP2K3 |  |  | 6986 | -0.064 | -0.0850 | No |
| 100 | DCAF11 |  |  | 7386 | -0.077 | -0.1206 | No |
| 101 | EZH1 |  |  | 7397 | -0.078 | -0.1114 | No |
| 102 | MBOAT2 |  |  | 7556 | -0.084 | -0.1184 | No |
| 103 | DCAF10 |  |  | 7668 | -0.089 | -0.1192 | No |
| 104 | TSPAN5 |  |  | 7726 | -0.092 | -0.1135 | No |
| 105 | CTSB |  |  | 7870 | -0.099 | -0.1167 | No |
| 106 | PIGQ |  |  | 8121 | -0.116 | -0.1300 | No |
| 107 | SLC11A2 |  |  | 8160 | -0.118 | -0.1186 | No |
| 108 | ENDOD1 |  |  | 8196 | -0.121 | -0.1065 | No |
| 109 | MINPP1 |  |  | 8308 | -0.133 | -0.1016 | No |
| 110 | NNT |  |  | 8335 | -0.136 | -0.0864 | No |
| 111 | LAMP2 |  |  | 8425 | -0.149 | -0.0767 | No |
| 112 | BSG |  |  | 8443 | -0.151 | -0.0584 | No |
| 113 | RBM5 |  |  | 8462 | -0.154 | -0.0399 | No |
| 114 | SLC2A1 |  |  | 8552 | -0.173 | -0.0270 | No |
| 115 | ATP6V0A1 |  |  | 8592 | -0.187 | -0.0065 | No |
| 116 | SLC25A37 |  |  | 8671 | -0.224 | 0.0144 | No |
Table: GSEA details [plain text format]

  

Fig 2: HALLMARK\_HEME\_METABOLISM: Random ES distribution      
 Gene set null distribution of ES for **HALLMARK\_HEME\_METABOLISM**
